# Our Bureau of Information

**Published:** 1911-11-25

**Authors:** 


### OUR BUREAU OF INFORMATION.

### Making Useful Progress.

THIS Bureau promises to be widely popular and useful. Correspondence is invited and suggestions still further to aid its work and development will be welcome.

### Wanted Experienced Honorary Correspondents.

We hope we made it clear in our issue of the 11th inst. that the position of Correspondent for the purposes of our Bureau is honorary, save that all out-of-pocket expenses to which a correspondent may be put in connection with the work thrown on him will, when necessary, be paid. We desire to form a Register of Correspondents, up and down the country, who may be willing to co-operate in the work of this Bureau. Applications, accompanied by a statement of past experience, and one or more references, should be sent to *The Editor*, "*The Hospital*," 29 *Southampton Street, Strand, London, W.C.*, marked "Bureau of Information" in the left-hand top corner of the envelope.

### Nursing and Private Homes, etc.

In reply to inquiries we have to say that owners of those establishments who are willing to provide accommodation for cases included in this page at certain rates may advertise in this section of the paper as and when vacancies may occur, providing they post at least two testimonials, one being from a practitioner of medicine, that the accommodation offered is satisfactory and the attendance good. No advertisements can be accepted for this department of the paper unless the foregoing rule has been observed and the home placed upon our List of Approved Accommodation for Invalids and Dependents. All letters relating to such homes should be addressed to The Manager, THE HOSPITAL'S Bureau of Information, 29 Southampton Street, Strand, London W.C.

### How to Make the Bureau a Success.

Everyone who uses or wishes to help our Bureau of Information must be kind enough to observe the following rules :

(1) Postal replies cannot be given. Exception will only be made in very special cases at the Editor's discretion.

(2) Queries or answers relating to different cases must be written, or preferably typed, on separate sheets of paper, and the writing must never be crossed.

(3) Questions and replies received not later than Wednesday will, as far as possible, be dealt with in our issue of the following Saturday week.

(4) Letters must have attached the name and address of the sender, with a pseudonym for publication if preferred.

(5) *All communications* for this department *must* be *accompanied by the coupon* to be found at the bottom of the third page of the cover on the inside, and should be addressed to the Editor of the Bureau of Information, c/o THE HOSPITAL, 29 Southampton Street, Strand, London, W.C.

*Notice.*—We invite workers of special experience and knowledge to offer help in replying to questions relating to their special department.

### Help Wanted and Found.

(1) A DEPENDENT OF SIXTY-EIGHT.

"A SON" writes : I am desirous of finding a home for my father where he can retain his liberty and a fair amount of privacy in or near London. He suffers from wasting of muscles and dresses with great difficulty, and his movements are very slow and somewhat uncertain. His intellect is quite clear, but he requires a lot of assistance now, and will shortly, I fear, be quite dependent on other people. Age, I think, sixty-eight. I will pay, say, 15s. a week and find him in pocket money for clothes and tobacco.

(2) HOME FOR OLD GENTLEMAN OF SEVENTY-TWO.

"CROMFORD" writes : I am taking advantage of your kind offer, and am writing to ask your advice about finding a "Home" for an old gentleman of seventy-two. He has been suffering from loss of memory and mental infirmity, and is now threatened with paralysis, and requires more nursing than is possible to give him in a small flat. He is a gentleman by birth and education, and, although ill and feeble, still retains all the instincts of a gentleman, so that we are very loth to place him in an ordinary asylum. He is in receipt of a 5s. pension, but has no other means, all having been spent upon him. Is there any charitable institution which would receive a case like this? We should be very glad of any information.

SUGGESTED HOMES FOR NOS. 1 AND 2.

Mr. Godfrey H. Hamilton writes : Patient might be taken temporarily at Country Branch of the National Hospital for the Paralysed and Epileptic. Apply to Secretary, Queen Square.

Other addresses : Co-operative Sanatoria, Mill House, Billericay, Essex (apply to Resident Secretary); Miss White, 48 Westbourne Gardens, Hove; Miss Canning, Seadown, Farncombe Road, Worthing; Nightingale Nursing Home, Hanley Road, Hornsey, N.

(3) HOME WANTED FOR DEFECTIVE BOY.

"LATE HOSPITAL MATRON" writes : Can you tell me of a Home, near Worcester preferred, where a boy of nine years could be received for about £1 1s. per week. He has not walked or talked since birth, and cannot sit up without support.

HOME FUND.

Mr. G. Hamilton, National Hospital for the Paralysed, writes : Application should be made to National Association for the Feeble-minded, 296 Vauxhall Bridge Road, S.W., who have a colony and could advise.

### Offers of Accommodation.

Every letter relating to this department, or to any case mentioned in it, must be accompanied by a coupon as provided in Rule 5, and also by copies of two good references or testimonials. We have again received offers of accommodation, not one of which is accompanied by a coupon or any references. They are accordingly disqualified and unavailable.

### Offers of Help.

All offers of this kind must be accompanied in every case by a coupon as provided in Rule 5. We have received some excellent replies and offers of help in the cases published already, for which we are most grateful.

